# Supplementary material for: Decrease of Pro-Angiogenic Monocytes Predicts Clinical Response to Anti-Angiogenic Treatment in Patients with Metastatic Renal Cell Carcinoma
Source: Cells. 2021 Dec 22;11(1):17. doi: 10.3390/cells11010017 (PMC8750389; doi:10.3390/cells11010017)
Supplement: Supplementary file 1 [file cells-11-00017-s001.zip › cells-1475493-supplementary.pptx]

## Slide 1
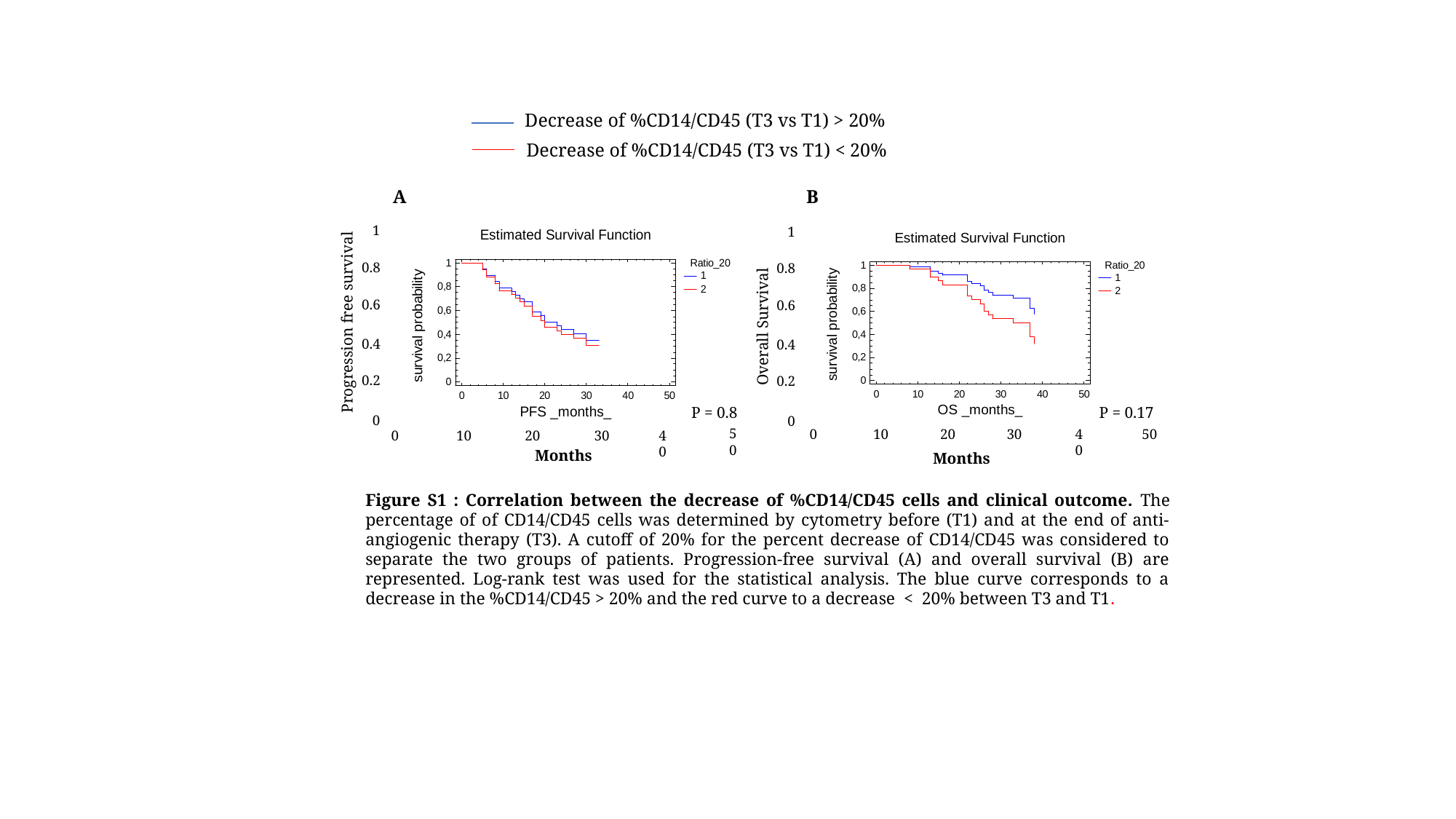

Decrease of %CD14/CD45 (T3 vs T1) > 20%
Decrease of %CD14/CD45 (T3 vs T1) < 20%
B
A
1
1
0.8
0.8
0.6
0.6
Progression free survival
Overall Survival
0.4
0.4
0.2
0.2
P = 0.17
P = 0.8
0
0
50
30
40
0
10
20
50
0
 10
20
30
40
Months
Months
Figure S1 : Correlation between the decrease of %CD14/CD45 cells and clinical outcome. The percentage of of CD14/CD45 cells was determined by cytometry before (T1) and at the end of anti-angiogenic therapy (T3). A cutoff of 20% for the percent decrease of CD14/CD45 was considered to separate the two groups of patients. Progression-free survival (A) and overall survival (B) are represented. Log-rank test was used for the statistical analysis. The blue curve corresponds to a decrease in the %CD14/CD45 > 20% and the red curve to a decrease < 20% between T3 and T1.

## Slide 2
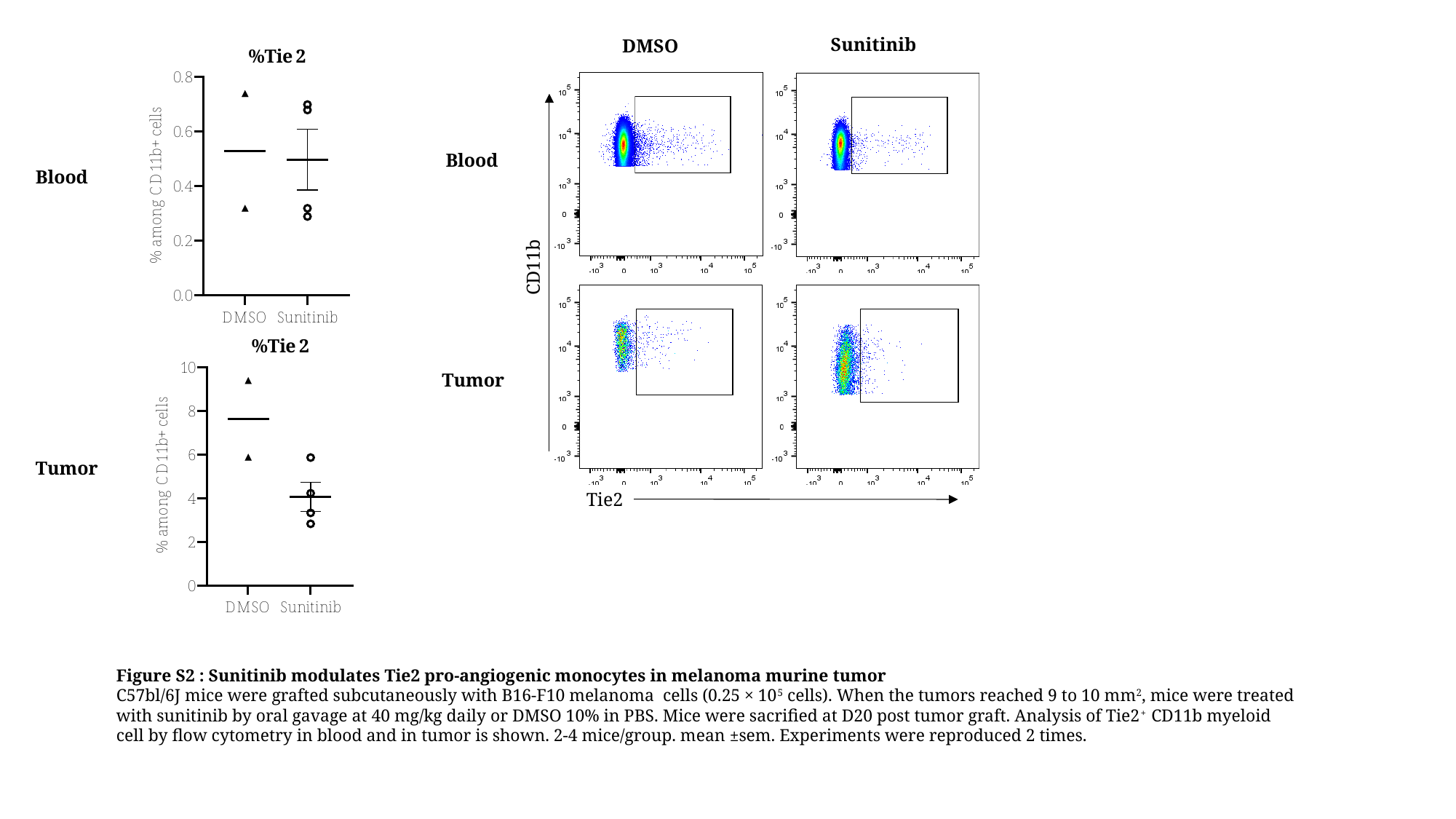

Sunitinib
DMSO
Blood
Blood
CD11b
Tumor
Tumor
Tie2
Figure S2 : Sunitinib modulates Tie2 pro-angiogenic monocytes in melanoma murine tumor
C57bl/6J mice were grafted subcutaneously with B16-F10 melanoma cells (0.25 × 10^5^ cells). When the tumors reached 9 to 10 mm2, mice were treated with sunitinib by oral gavage at 40 mg/kg daily or DMSO 10% in PBS. Mice were sacrified at D20 post tumor graft. Analysis of Tie2+ CD11b myeloid cell by flow cytometry in blood and in tumor is shown. 2-4 mice/group. mean ±sem. Experiments were reproduced 2 times.
